# Cystic lymphangioma of the upper limb: Clinical case and literature review

**DOI:** 10.1259/bjrcr.20210206

**Published:** 2022-09-12

**Authors:** Daouda Diarra, Younes Mekouar, Abdoulfatahi Salihou, Boubacar Traore, Dalale Laoudihy, Kamilia Chbani, Siham Salam, Lahcen El Ouzidane

**Affiliations:** 1 Department of Pediatric Radiology, CHU Ibn Rochd, Faculty of Medicine and Pharmacy of Casablanca, Hassan II University, Casablanca, Morocco; 2 Laboratory of Epidemiology, Faculty of Medicine and Pharmacy of Casablanca, Hassan II University, Casablanca, Morocco

## Abstract

We report an observation of a macro- and microcystic lymphatic malformation located in the right upper limb. This was a 5-year-old girl with no previous pathological history, followed since the age of 11 months for a congenital subcutaneous, painless and soft swelling of the right upper limb.

Ultrasound of the soft tissue and magnetic resonance imaging (MRI) allowed the diagnosis of macro- and microcystic lymphatic malformation of the right upper limb.

There is little epidemiological data on cystic lymphatic malformations (CLM). Superficial MLKs are more numerous than deep MLKs; of the superficial MLKs, nearly 75% are located in the head and neck, with an estimated incidence of 1.2 to 2.8 per 1000 births, and in the axillary hollows in 20% of cases. They affect equally males and females and different ethnic backgrounds. Involvement of the upper limb and particularly the arm is very rare.

MRI plays an important role in the diagnosis and assessment of the tumor’s boundaries.

Treatment can be difficult because of the location of the tumor and its extension into the surrounding tissue.

## Introduction

Cystic lymphatic malformations, formerly called cystic lymphangiomas, are rare, hemodynamically inactive, benign, mature lymphatic malformations consisting of abnormal lymphatic vessels and cysts of various sizes and shapes.^1–4^ The most commonly used classification divides them into microcystic, macrocystic and mixed lesions depending on whether the volume of the cystic spaces is less than or greater than 2 cm^3^^5,6^. Some authors prefer to classify them as capillary lymphangiomas, cavernous lymphangiomas and cystic hygromas.^7^

Macrocystic lymphatic malformations (MLMK), also known as cystic lymphangiomas or cystic hygromas, are a circumscribed variant of deep lymphangiomas that expand easily. They are usually located in the cervico-facial or axillary region and more rarely in the mediastinum or retroperitoneal region. Rarely, they are located in the limbs.^8^

On the other hand, microcystic lymphatic malformations may present as swellings under normal skin colour. They tend to progress over time and often induce deformities with functional repercussions depending on the topography. They may also be superficial and epidermal.^9^

We report an observation of cystic lymphangioma of the right upper limb.

## Clinical presentation

She was a 5-year-old girl with no pathological history, from a non-consanguineous marriage, with good psychomotor development, followed since the age of 18 months for a congenital subcutaneous swelling of the right upper limb progressively increasing in volume.

The initial clinical examination revealed a right anterolateral thoracic subcutaneous swelling, right axilla extended to the right arm and forearm. On palpation, the swelling was painless, soft, and non-throbbing. The rest of the examination was unremarkable.

MRI showed a large formation containing multiple cystic pockets of varying size, some of which were macrocystic in the anterior chest wall, posterior and external arm wall and forearm, and others of which were microcystic in the axillary fossa and inner arm.

**Figure 1.**
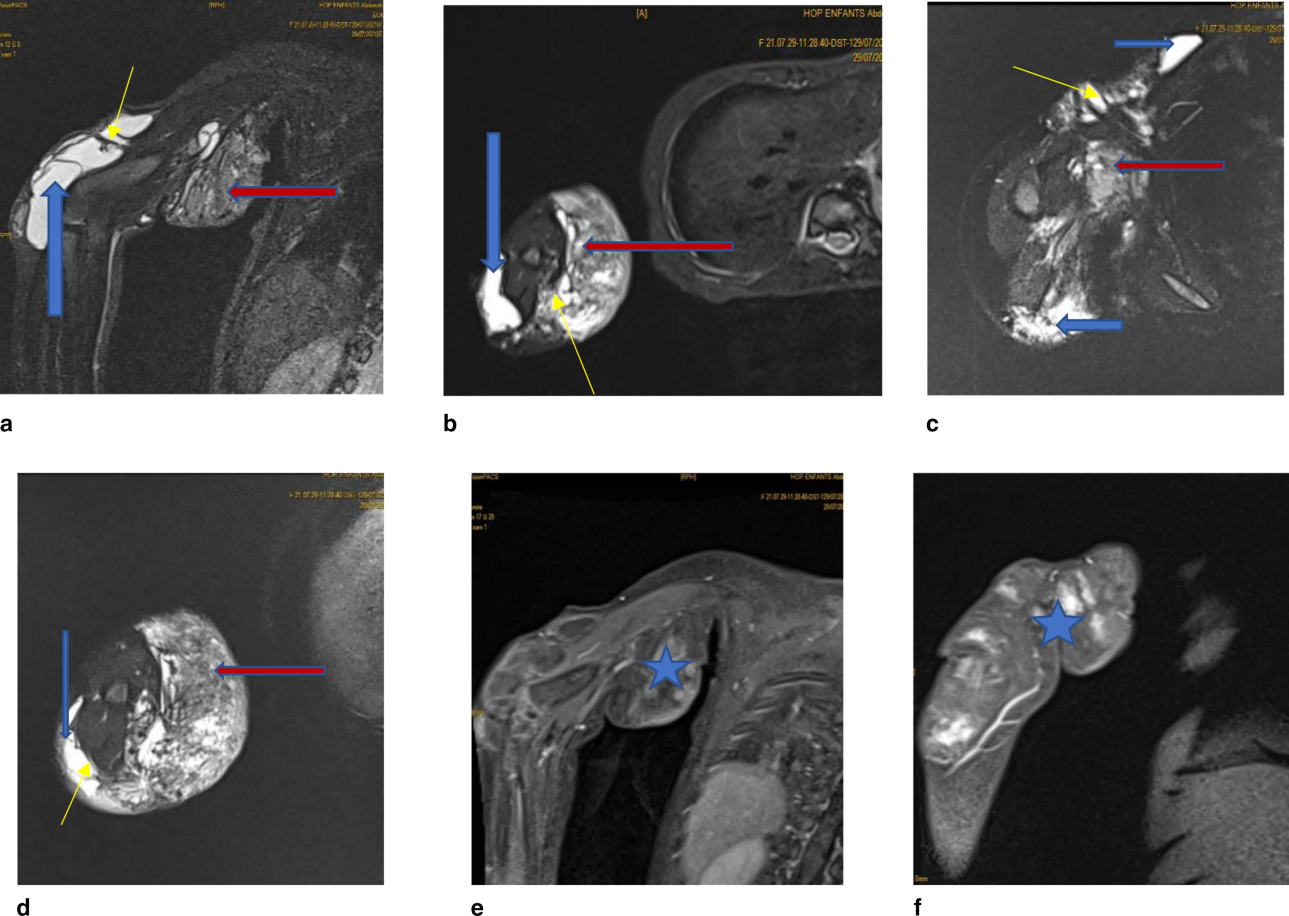
MRI of the right upper limb: cystic lymphangioma of the right axillary soft tissues extended to the arm and the upper third of the forearm with vascular involvement and extension to the thorax. (a) coronal TIR sequence, (b) axial sequence STIR, (c) axial T2 FATSAT sequence, (d) axial T2 FATSAT sequence, (e) and (f) T1 sequence with gadolinium injection. Formation containing multiple cystic logettes of pure liquid signal, of variable size, some of which are macrocystic (red arrows) and others of microcystic appearance (blue arrows). They have hyposignal septa (yellow arrows) on all sequences. They are mostly subcutaneous with intra-muscular extension and encompassing of the vascular pedicle (blue stars) in the axilla and humerus.

They have a pure fluid signal (T1 hypo signal and T2 hyper signal) with the septa showing an intermediate signal on T1, T2 and STIR sequences (Figure 1).

They are mostly subcutaneous with intramuscular extension to the pectoralis major and the posterior part of the arm with enclosure of the vascular pedicle in the axilla and humerus.

MRI was used to diagnose cystic lymphangioma of the right axillary soft tissue extending to the arm and upper third of the forearm, of macro- and microcystic type with vascular involvement and extension to the thorax.

## Traitement and evolution

The proposed therapeutic strategy was multidisciplinary. A partial and wide surgical resection was performed. Examination of the surgical specimen showed a poorly limited lesion with cystic cavities; the histological study found a proliferation of lymphatic vessels without angiomatous component. The lateral and deep boundaries were unclear. The immediate postoperative course was simple with no significant complications.

## Discussion

Cystic lymphomas arise during embryonic development when the primary lymph sacs fail to fuse with the central venous system. They are round or lobulated and compressible masses.

Their incidence is approximately 1 per 12,000 births. They are present at birth in 30 to 50% of cases and are discovered in 90% of cases before the end of the second year of life.^1^ They rarely appear in adulthood.

The pathogenesis of MLMK is incompletely elucidated. However, advances have been made thanks to molecular biology. Indeed, many regulatory genes such as vascular endothelial growth factor receptors, VEGFR3 and VEGFR2, whose ligand is VEGF-C, have been implicated in the pathogenesis. These receptors are involved in the growth of lymphatic vessels in the skin without influencing the development of blood vessels.^10,11^ On the other hand, endothelial cells in cystic lymphangiomas have been found to secrete a significant amount of basic fibroblast growth factor (bFGF), which can be considered an inducer of angiogenesis. The level of thrombospondin-1 (an inhibitor of angiogenesis) secreted by the cells of lymphatic malformations is lowered in parallel. PMLKs will, therefore, be derived from these angiogenesis inducers.^12^

MLMKs are in the neck in 75% of cases and in the axillae in 20% of cases, with the possibility of communication between these two locations via bridges passing under the clavicle. They are in the mediastinum, the retroperitoneum or the pelvic region in 5% of cases.^4^

Involvement of the upper extremity, particularly the arm, is very rare^13,14^ Indeed, a few rare cases of branchial localization (2/19 patients) and limb involvement (8/72 patients), including two (2) in the upper extremity, have been found in patients with cystic lymphangiomas in the literature.^15,16^

The diagnosis of MLMK is made in view of the clinical appearance of the lesions, which are soft, lobulated, renal swellings, not attached to the underlying skin, and not very mobile in relation to the deeper layers.^1^

Macrocystic and microcystic components evolve differently over time^1,9^

Macrocystic lymphatic malformations may regress after inflammatory flare-ups, especially with proximal infections, or after intracystic hemorrhage, which induces “natural sclerosis”. In contrast, microcystic components tend to progress over time, becoming thicker and more troublesome.^1,9^

Microcysts may be superficial or epidermal and present as translucent or hemorrhagic millimetric vesicles, scattered or grouped in patches, called lymphangiectasias.^17^ They may be complicated by oozing (lymph) and bleeding when covered by verrucous hyperkeratosis, which can lead to long-term iron deficiency anaemia.^1^

The appearance of the lymphangiectatic area may become purple or black, following blood clotting, with a spontaneous evolution towards progressive aggravation associated with an increase in the number of lymphangiectatic vesicles, thickening, increased oozing and bleeding.

These eroding lymphangiectasias constitute a skin breach and may become an infectious portal of entry. Underlying bacterial cellulitis or even bacteremia or severe sepsis may result.

Profound discomfort (due to the unsightly appearance of lymphangiectasias), oozing and bleeding, as well as the malodorous character associated with bacterial colonization, are also associated with lymphangiectasias.^2,3^

Ultrasound is of interest for positive and sometimes differential diagnosis. It shows multilocular cystic masses with septa of variable thickness. The contents are anechoic, hypoechoic or hyperechoic, depending on whether the lymphatic fluid is infected, haemorrhagic or hyperlipidic.^18^

MRI highlights the characteristic aspect of hyposignal in T1 and hypersignal in T2. It also allows a better assessment of the extension of the tumour in different sections (sagittal, axial and coronal) and the involvement of adjacent structures that are not clinically suspicious, thus providing a valuable aid to surgery.^19^

### Treatment

Therapeutically, several means are available such as sclerotherapy, surgery, laser and radiotherapy. The indication depends essentially on the micro- or macrocystic type of the lymphatic malformation and its anatomical location.^1^

Sclerotherapy is the first-line technique for disabling macrocystic lymphatic malformations.

It is less effective in microcystic lymphatic malformations. Depending on the availability of products, the experience of the practitioner, the macro- or microcystic character and the location of the malformation, different sclerosing agents can be used (hypertonic saline, lipiodol, bleomycin and boiling water). Ethibloc, the most commonly used sclerosing agent in France, appears to be successful in the treatment of MLMK.^20^ This agent polymerises rapidly on contact with the blood and appears to produce biodegradable emboli that are resorbed within 4 to 6 weeks after injection. It thus causes a gigantocellular reaction necessary for the walls of the cyst to collapse. This hardens and disappears in 2 to 6 months.^20^ The most frequent complications are febrile inflammatory reactions, inflammatory nodules and oozing ulcerations.

The results of this procedure are excellent or good in 60% of cases. Some complications, such as hemoglobinuria, are reported in up to 50% of patients after sclerotherapy with absolute ethanol. Overall, the risk of significant complications such as nerve damage, skin necrosis, pulmonary vasospasm, cardiac arrhythmia or cardiopulmonary collapse is in the order of 0 to 3%.^21^ Absolute ethanol is the sclerosing agent with the highest rate of serious complications.

### Surgery for cystic lymphatic malformations is a second-line technique

Complete surgery limits the risk of recurrence, but it is rarely feasible. On the other hand, partial surgery may be indicated to reduce the volume of a large microcystic or mixed lymphatic malformation.

Removal of macrocystic lymphatic malformations can sometimes be difficult because of the anatomical location of the lesion, or its extension. The parotid region is the most difficult to approach. Thus, the complete removal of lymphangiomas sometimes requires several procedures.^9^

Lasers and radiofrequencies are useful for superficial lymphangiectatic components of cystic lymphatic malformations but must be performed by an experienced operator. They are safe and effective in the short term but are suspensive. Very painful is that they are often performed under general anesthesia,^9^

### Physiotherapy techniques

Compression and manual lymph drainage have not been proven to be effective in cystic lymphatic malformations. They can be proposed and continued on a case-by-case basis, depending on the potential benefit for each patient.^9^

## Conclusion

Cystic lymphatic malformations can be symptomatic from the prenatal period or later in life, most often in childhood. The distinction between macrocystic, microcystic and mixed lymphatic malformations conditions the prognosis and the therapeutic approach.

This observation is original because of the unusual location and the macro- and microcystic type of the cystic lymphatic malformation. Moreover, it raises the therapeutic difficulties of these malformations. In first intention, macro-cystic lymphatic malformations are more likely to be treated by sclerotherapy, but surgical treatment should not be ruled out, especially in microcystic malformations.

## Learning points

- The distinction between macrocystic, microcystic and mixed lymphatic malformations conditions the prognosis and the therapeutic approach.
- Microcystic or mixed lymphatic malformations evolve over time with a tendency to progressive worsening.
- Ultrasound and MRI are the two imaging examinations indicated to confirm the diagnosis and to assess the extension of a cystic lymphatic malformation.
- The management of microcystic or mixed lymphatic malformations is more complex and is based on therapeutic strategies decided in multidisciplinary consultation, which may include sclerotherapy, physiotherapy, surgery, lasers, sirolimus, targeted therapy, successively or in combination, with windows of therapeutic abstention.
